# Optimizing placement of constructed wetlands at landscape scale in order to reduce phosphorus losses

**DOI:** 10.1007/s13280-020-01349-1

**Published:** 2020-09-12

**Authors:** Faruk Djodjic, Pia Geranmayeh, Hampus Markensten

**Affiliations:** Department of Aquatic Sciences and Assessment, Swedish University of Agricultural Sciences, Lennart Hjälmsv. 9, P.O. Box 7050, 75007 Uppsala, Sweden

**Keywords:** Constructed wetlands, Modelling, Optimised placement, Phosphorus retention

## Abstract

Constructed wetlands (CWs) are one of the main countermeasures to reduce diffuse phosphorus (P) losses, but there is still a lack of systematic guidance accounting for spatially variable effects of hydraulic and P load on P retention. We present a three-step modelling approach for determining suitable placement of CWs in four different size groups (0.1–1.0 ha), based on incoming hydraulic and P load. The modelled hypothetical CW area was only 17% of that previously estimated and area of efficient CWs is even lower. The mean area-specific P retention increased with CW size. However, the spatial variation in retention was large for all size groups and largest (6–155 kg ha^−1^ year^−1^) for the smallest CWs due to highly variable incoming P loads, showing the possible benefits of targeted placement of CWs. The presented modelling approach has also flexibility to include and account for possible future changes in land cover and management.

## Introduction

Following successful reductions in nutrient loads from point sources such as wastewater treatment plants, agriculture is now considered to be the main non-point source of eutrophying nutrients in many parts of the world (Carpenter et al. 1998; Sharpley et al. 2015). In Sweden, agriculture is estimated to be the largest anthropogenic source of both nitrogen (N, 23 300 t) and phosphorus (P, 460 t) (Ejhed et al. 2016). Construction or restoration of wetlands is an important countermeasure to reduce nutrient delivery to aquatic ecosystems (Fisher and Acreman 2004; O’Geen et al. 2010). Since 2010, approximately 4500 hectares of new wetlands have been constructed or restored in Sweden, with on average of more than 500 ha of new wetlands constructed annually, at a cost of approximately 30 million SEK per year (Swedish Environment Protection Agency 2019). As intensification of mitigation efforts to reduce nutrient losses from agriculture is required, further increases in the number and area of wetlands can be expected. For instance, the Swedish government will invest ~ 200 million SEK in the construction/restoration of wetlands in the period 2018–2021.

The P removal efficiency of CWs receiving runoff from non-point sources varies considerably, between 1 and 88% (Braskerud et al. 2005; Kynkäänniemi 2014), with a mean value of 33% and a median value of 26% (Kynkäänniemi 2014). A systematic review of European, Asian, and American CWs showed that the median removal efficiency of total P (TP) was 44% for CWs treating agricultural runoff. However, CWs with precipitation-driven flow had lower removal efficiency, 21% TP (Land et al. 2016). The P removal efficiency varies depending on wetland design, location, annual variation in water flow, and P loading (Braskerud et al. 2005; Tonderski et al. 2005; Kynkäänniemi et al. 2013; Land et al. 2016). Hence, it is important to consider these factors during planning of future wetlands to make them as efficient as possible. Recently, Ulén et al. (2019) showed that it is possible to achieve high TP retention efficiency (36%) in a Swedish CW located in a critical source area (CSA) and specially designed to trap P. At present, there are recommendations covering the planning phase in construction of new wetlands, concerning e.g. the wetland to catchment area ratio (as a proxy for the hydraulic load (HL)) and the land use distribution in the wetland catchment (as a proxy for P load). However, there are no explicit guidelines on optimisation of CW location, size and P retention capacity to increase nutrient retention efficiency in general and P retention in particular. Studies have shown that an increase in wetland to catchment area ratio increased the P retention efficiency, while the area-specific retention (kg ha^−1^ wetland area) decreased (Uusi-Kämppä et al. 2000). Furthermore, Kynkäänniemi (2014) showed a strong positive linear correlation (*R*^2^ = 0.78) between HL and annual P accumulation up to a HL threshold of approximately 120 m year^−1^. It has been found that a HL of 300 or 400 m year^−1^ can have a detrimental effect on annual P accumulation and should be avoided (Johannesson et al. 2015). Consequently, the full P reduction potential of CWs cannot be achieved at too low or too high hydraulic and nutrient loads. On the one hand, CWs may be oversized and there may not be enough incoming nutrients to exploit the full reduction potential, leading to low cost-efficiency. On the other hand, too high HL may cause the residence time to be too short to allow retention processes to be effective (Koskiaho 2006). Modelling water flow pathways at catchment scale using digital elevation models (DEMs) and GIS-based soil hydrology classifications offers a useful template to identify and rank vulnerability to erosion and overland flow (Sharpley et al. 2015). We devised a three-step approach for optimising CW placement in the landscape. The first step is to calculate annual hydraulic load in a catchment, based on measured or modelled water discharge, high-resolution flow pathways and assumed size of the CW. Current developments in terms of growing access to high-resolution data and modelling approaches have enabled accurate identification of CSAs at landscape and catchment scales (Thomas et al. 2016; Djodjic et al. 2018). Using a modelling approach and high-resolution data, erosion risk maps were recently developed for the southern half of Sweden, covering more than 90% of Swedish arable land (Djodjic and Markensten 2018). In the second step of our approach, following calculation of water volumes and HLs reaching the CWs, nutrient loads entering the CWs are calculated. Nutrient emissions from non-point sources, especially P emissions, vary greatly between and within arable fields. The majority (80%) of P losses originate from a small proportion of catchment areas (20%), a situation known as the 80:20 rule (Sharpley et al. 2009). Therefore, abatement measures spatially targeted at locations where pollution is substantial have potential to be more effective (Sidemo-Holm et al. 2018). In the third step, potential P retention can be calculated as a function of incoming P loads (Weisner et al. 2016), based on the calculated P load in the second step. Phosphorus retention and reduced eutrophication of aquatic recipients can then be achieved by increasing the amount of lost nutrients accumulating in a CW close to the source, and maintenance by dredging and returning nutrients to arable fields will also be more cost-efficient. As the efficiency of CWs is highly dependent on the incoming loads, which in turn are governed by upstream land cover and management, any structural changes in bio-resource economy (bioeconomy) leading to changed land cover and management may alter the efficiency of CWs. Rakovic et al. (2020) describes the alternative development paths with a focus on changes in land cover and management in the Nordic region in the year 2050—the Nordic Bioeconomy Pathways (NBPs). Therefore, any robust methodology for optimal placement of CWs needs to be flexible and able to account for such alternative paths and possible changes.

In this study, we used high-resolution (2 m × 2 m) distributed modelling to calculate the optimal placement of CWs as a function of incoming water volumes and P loads. Specific objectives were (i) to reliably estimate the optimal number and total area of effective CWs of various sizes at catchment scale, (ii) to quantify the highly variable P retention potential of targeted CWs and (iii) to compare and prioritise the most effective CWs at catchment scale.

## Materials and methods

### Case study catchments

**Fig. 1:**
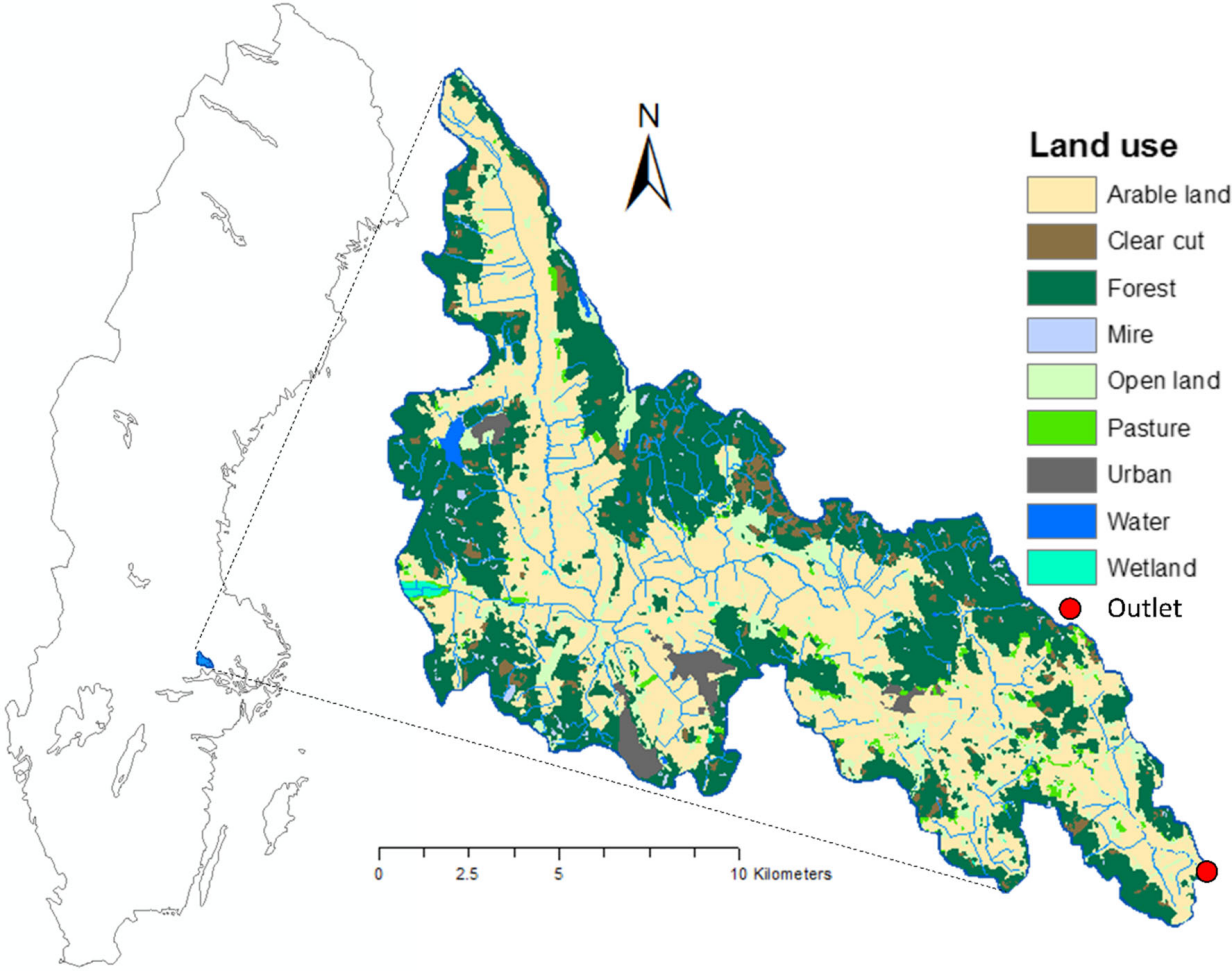
Maps showing the location of the study catchment, Lillån, in central Sweden and land uses in the catchment

Lillån catchment is a medium-large (192 km^2^) catchment situated in central Sweden (Fig. 1). Forest (38%), generally covering upper parts of the catchment, and arable land (45%), in the Lillån river valley, are the dominant land use categories (Fig. 1). Silty clay (34%) and silty clay loam (11%) are the dominant soil textural classes on arable land, while glacial till (20%) is the dominant soil type on forested land. Based on water flow measurements for 1978–2018 at the Gränvad gauging station (situated within the Lillån basin and with a sub-catchment of 167.5 km^2^), mean annual discharge is 234 mm. The general flow direction of Lillån is from the north-west to the outlet in the south-east (Fig. 1). According to Water Information System Sweden (WISS), there are already 8 CWs (area range 0.9–29 ha, average 9 ha) covering a total area of 71 ha in Lillån catchment, but there are no P ponds (Water Information System Sweden 2020). The Lillån catchment was chosen as a case study in the present analysis since it has been used previously to exemplify the estimated abatement potential of different countermeasures, including wetlands and P ponds, by water authorities (Gyllström et al. 2016). Since then, it has been used as a template for similar estimations in other river basins across Sweden. Phosphorus ponds are usually small wetlands situated in upstream parts of a catchment, close to arable land (Braskerud et al. 2005).

Since there are no adequate measurements of water chemistry in Lillån catchment, two small catchments dominated by arable land, C6 and U8, in the vicinity of Lillån were used to compare modelled P loads with measured P loads in the present study. Both catchments are part of the national environmental monitoring programme, which includes a total of 21 small agricultural catchments (area < 35 km^2^), some of which have been monitored since the 1990s with the focus on nutrient losses (Kyllmar et al. 2014). We selected a study period of eight agro-hydrological years (June 2008 to July 2016) for which calculated TP load data, based on flow-proportional collection of water samples, were available for both catchments. Data on transported monthly loads of P for both catchments were downloaded from a database held at the Department of Soil and Environment, Swedish University of Agricultural Sciences (SLU 2018). Catchments C6 and U8 are situated just 20 km east and south-west of Lillån catchment, respectively, and have comparable soil textural composition dominated by clay soils. The exact location of these catchments cannot be revealed, due to the policy of the national monitoring programme. Catchment C6 occupies an area of 33.1 km^2^, of which 59% is arable land, whereas catchment U8 is smaller (5.7 km^2^) and has 56% arable land.

### Identifying optimal CW locations

As mentioned above, Kynkäänniemi (2014) reported a strong positive linear correlation (*R*^2^ = 0.78) between HL and annual P accumulation in CWs up to a threshold of approximately HL = 120 m year^−1^. Based on this, in the first step of our modelling process, we identified optimal locations for potential CW with the optimum HL of 100 m year^−1^, in order to include a small uncertainty margin to the threshold value of 120 m year^−1^. The hydraulic load was estimated by dividing the annual flow volume entering each CW (m^3^) with the CW area. The annual flow was estimated based on specific runoff. We used measured long-term annual average of 234 mm for the whole Lillån catchment and modelled flow accumulation. The basis for the modelling work was a DEM in raster format. A 2-m grid based on LiDAR data was used, with a density of 0.5–1 point m^−2^ and accuracy usually better than 0.1 m (Lantmäteriet 2014). Calculations of flow direction and flow accumulations were performed using PCRaster software for environmental modelling (Schmitz et al. 2009). When necessary, obvious errors in the DEM, such as road culverts, were corrected. First, based on measured mean annual-specific runoff (234 mm) and flow accumulation lines, annual flow in m^3^ was calculated for all grid cells. Second, based on four different CW sizes (0.1, 0.2, 0.5 and 1.0 ha), covering the range of sizes of CWs used in Sweden, optimal locations in the landscape were identified, where HL = 100 m ± 10%, for each of the four different sizes of CWs.

### Modelling P load

In the second step, after calculating the optimal location with a hydraulic load of approximately 100 m year^−1^ for each CW, the P load entering each CW was calculated. We modelled P load using a combination of distributed modelling and export coefficients (Johnsson et al. 2016) produced using the ICECREAM model (Larsson et al. 2007). The same flow direction and flow accumulation maps as in the first step were used. Results obtained using the ICECREAM model have long been used as the basis for estimating TP losses from arable land in Sweden (Johnsson et al. 2008, 2016, 2019). They are also used in the calculation of nutrient loads to the Swedish marine environment, i.e. in Pollution Load Compilation (PLC) (Ejhed et al. 2016). In short, ICECREAM calculates P losses based on a set of parameters characterising the production system, including geographical region, and representing climate, agricultural management and production, crop distribution, soil textural distribution, soil P content and field slope. Sweden is divided into 22 leaching regions, which differ regarding climate, crop yield and management operations. The latter are covered by data on fertilisation and manuring, ploughing, sowing, harvesting etc. collected by Statistics Sweden. Further, arable soils are divided into 10 textural classes and all crops exceeding 1% of the total arable land in each leaching region (in total 15 crops) are included in the crop rotation sequences. Based on crop distribution, crop sequences covering a modelling period of 15,000 years are created for combinations of each leaching region and each soil textural class (Johnsson et al. 2019). This is done by repeating a climate time series covering a 30-year period. Thereafter, based on all years, when a certain crop is modelled in the 15,000-year crop sequence, average annual values, i.e. export coefficients, of N and P losses are calculated for every combination of crop, soil and leaching region, and average export coefficients are calculated for all arable crops. These long crop sequences are needed to obtain a sufficient number of occurrences of all crops to secure reliable estimations of average values normalised for weather conditions. In this study, due to lack of data on exact crop distribution on field level, the mentioned average value for arable crops was used for arable land. All export coefficients used in this study are representative of leaching region 6, in which all modelled catchments (C6, U8 and Lillån) are situated. The export coefficients for other land use categories, such as forest (0.013 mg l^−1^), clear cuts (0.021 mg l^−1^) and open land (0.026 mg l^−1^), were determined according to HELCOM’s Sixth Pollution Load Compilation (Ejhed et al. 2016). In the distributed model, each grid cell was assigned a representative export coefficient based on land use category, with the exception of arable land. In the case of arable land, the representative export coefficient was assigned based on the soil map of textural classes of Swedish agricultural soils (Söderström and Piikki 2016; Piikki and Söderström 2019). Specific runoff (mm, i.e. l m^−2^), as either measured average annual (Lillån) or measured monthly values (C6 and U8), was then multiplied by the export coefficients (mg l^−1^) to calculate loads. Calculated loads for each grid cell were accumulated along the flow pathways to illustrate both area-normalised flux (kg km^−2^) and total flux (kg). There are no systematic measurements of water quality in the Lillån catchment. Therefore, in order to test the applicability of our new approach, dynamic modelling of P load on monthly time steps was performed for a period of eight agro-hydrological years (July 2008 to June 2016) for catchments C6 and U8 and compared with measured monthly loads. Measured monthly discharge at the outlets of C6 and U8 was used as a driving variable in the dynamic model, where it was taken as specific runoff and multiplied by the land use-/soil-specific export coefficients to calculate loads. The modelled loads for each grid cell were thereafter accumulated along the calculated flow pathways and the accumulated load at the outlet cell was recorded on monthly steps, exported and compared with the measured loads. Model performance and goodness of fit were estimated by visual comparison, coefficient of correlation, Nash–Sutcliffe coefficient (Nash and Sutcliffe 1970) and the ratio between modelled and observed TP losses, expressed as a percentage. Based on the optimal location of CWs calculated in the first step and the P loads accumulated along the flow pathways in the second step, a CW-specific P load reaching each potential CW was extracted.

### Modelling P retention

Based on measured data from 15 wetlands, Weisner et al. (2016) developed a polynomial second-degree function (*R*^2^ = 0.70) to estimate annual P retention as a function of annual P load:

$${{Pret}} = - 0.0003 \times ({{Pload}})^{2} + 0.4584 \times {{Pload}}$$

where *Pret* (kg ha^−1^ year^−1^) is P retention and *Pload* (kg ha^−1^ year^−1^) is the modelled P load. In the third and final step of our approach, this equation was used together with the modelled P loads reaching each potential CW to calculate potential CW-specific P retention.

## Results

### Optimal location of CWs of different sizes

**Fig. 2:**
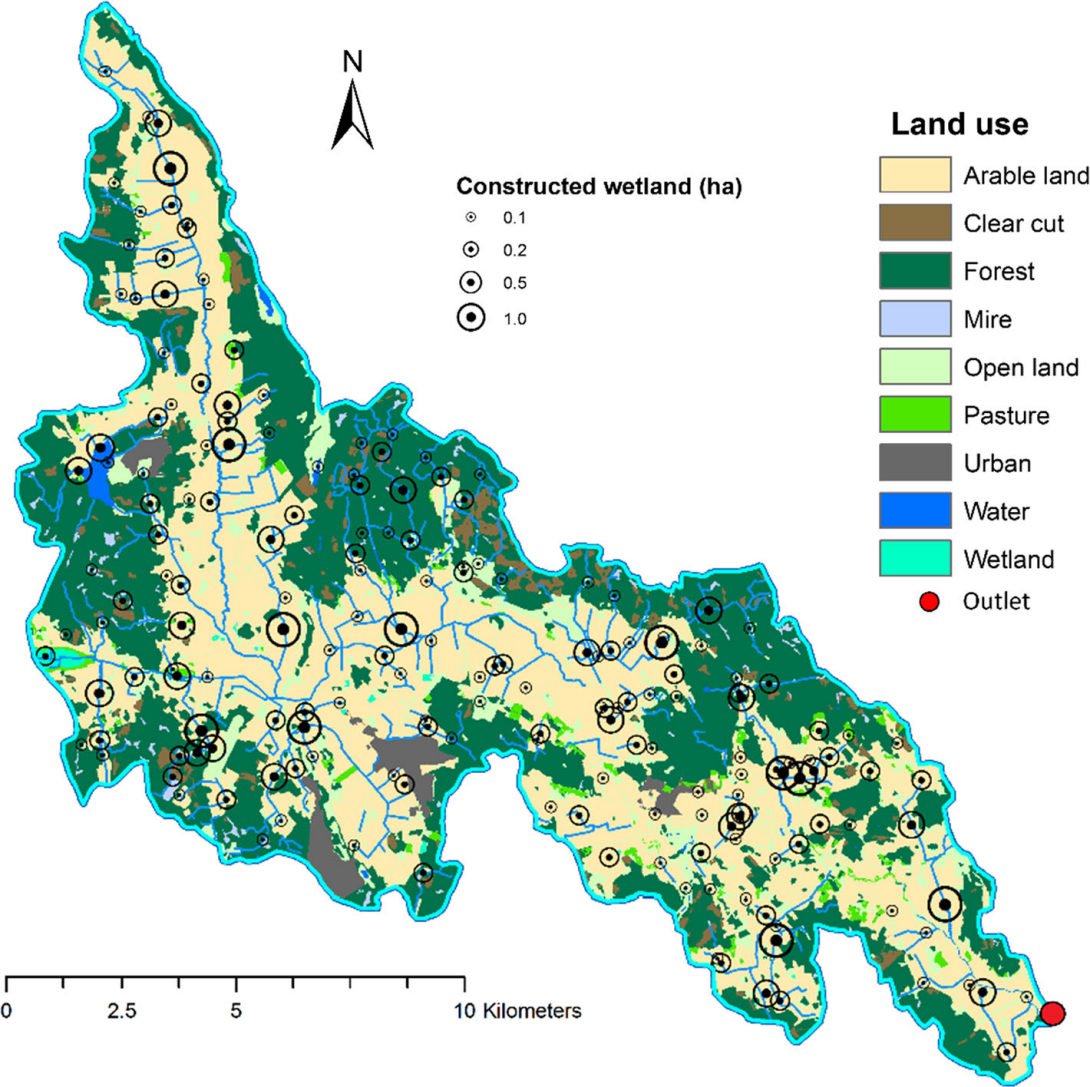
Distribution of potential constructed wetlands of four size groups (0.1, 0.2, 0.5 and 1.0 ha) in Lillån catchment

Calculations of the HL and optimisation of the placement of CWs in four different size groups resulted in a total of 191 potential CWs (11 CWs of 1.0 ha, 23 CWs of 0.5 ha, 59 CWs of 0.2 ha and 98 CWs of 0.1 ha) in the Lillån catchment. Adding up the values for all these 191 CWs, which met the criteria of HL = 100, in all four groups revealed potential for a total CW area of 44.1 ha in Lillån catchment. The geographical distribution of these potential CWs is shown in Fig. 2. Although the majority (125 out of 191 CWs) of all CWs were located on arable land, approximately 34% were still placed in other land use categories, mostly forest. The calculated catchment area of potential CWs in Lillån catchment varied between 43 and 427 ha, for 0.1-ha and 1.0-ha CWs, respectively.

### Modelling P loads

**Fig. 3:**
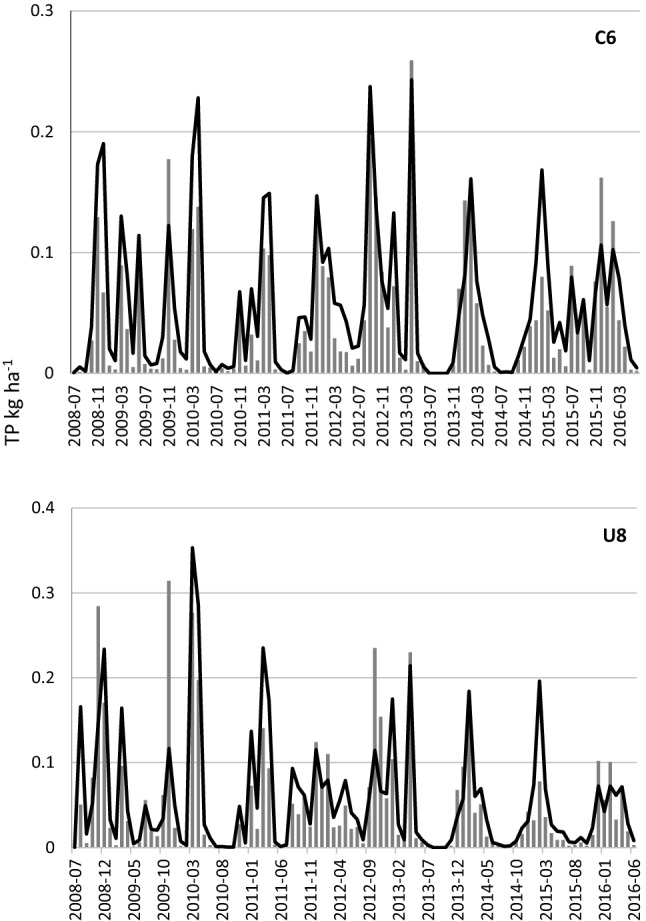
Measured (bars) and modelled (line) loads of total phosphorus for the two agricultural catchments (C6 and U8) representing the study catchment

The measured and modelled loads of TP for catchments C6 and U8 are shown in Fig. 3. Coefficient of correlation (*R*^2^) between measured and modelled loads at monthly time steps was 0.81 and 0.66 for C6 and U8, respectively, indicating good agreement in monthly dynamics between measured and modelled loads. The corresponding value of the Nash–Sutcliffe coefficient was 0.70 for C6 and 0.60 for U8. The ratio between modelled and observed TP losses was 131% for C6 and 114% for U8, indicating overestimation of modelled values compared with measured loads, through the model overestimating some high-flow episodes (Fig. 3).

Total annual P load from the Lillån catchment was estimated to be 10,405 kg, resulting in mean area-specific annual load of 0.54 kg P ha^−1^ year^−1^. The corresponding measured values for catchments C6 and U8 during the period June 2008 to July 2016 were 0.50 and 0.59 P ha^−1^ year^−1^, respectively, reflecting similarities in land use and soil textural distribution and their effects on P losses.

### Calculations of potential P retention in CWs

**Fig. 4:**
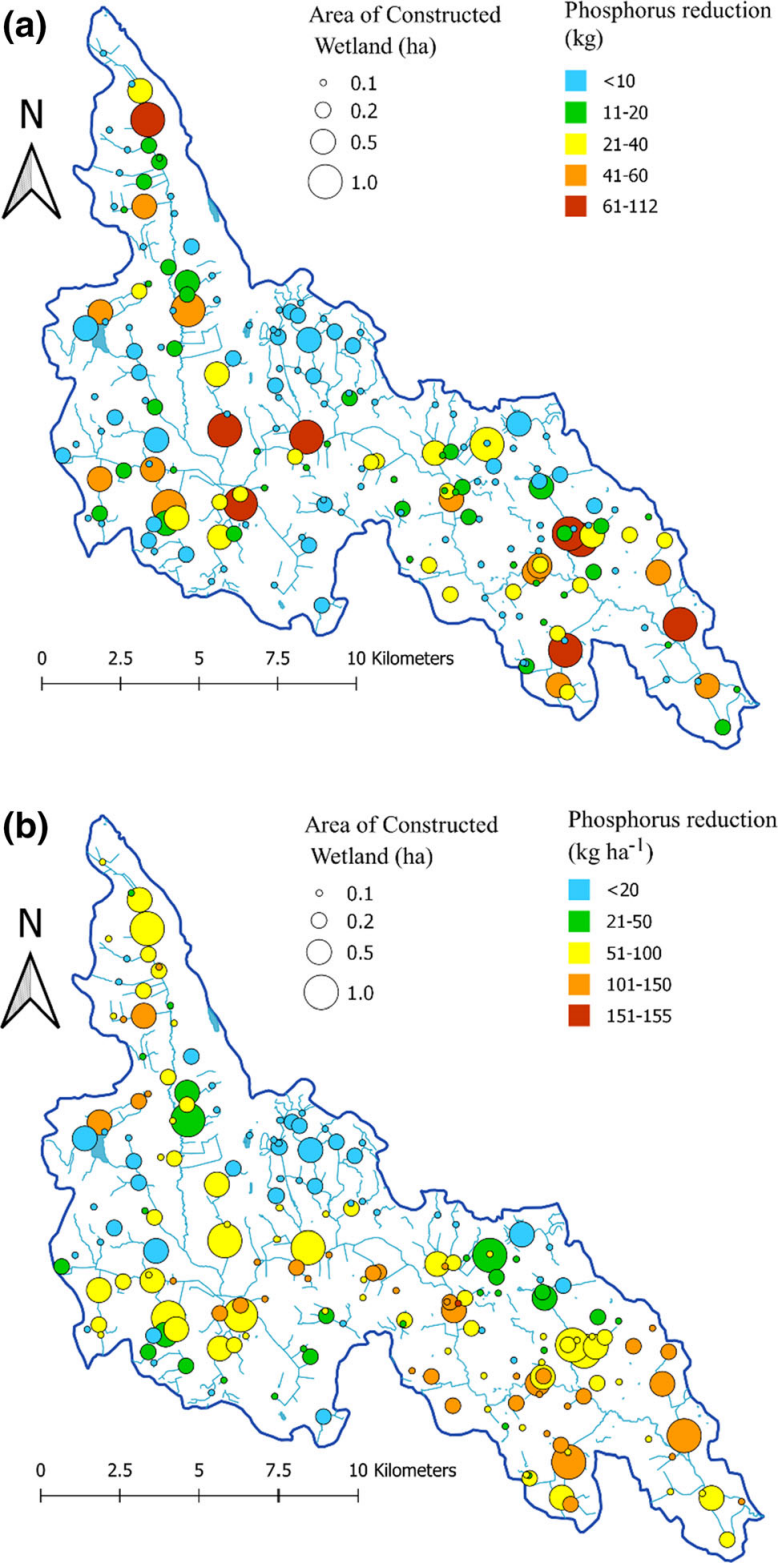
**a** Reduction in total phosphorus (TP) expressed as total amount (kg) and **b** as area-specific reduction (kg ha^−1^) per constructed wetland (CW) of four different sizes. Size of the circles shows the CW area, whereas colours illustrate TP reduction

Figure 4 visualises the potential P retention calculated for the hypothetical 191 CWs of different sizes in the Lillån catchment. Figure 4a shows the total P reduction (kg) and Fig. 4b the area-specific P reduction (kg ha^−1^) for each CW. As expected, larger CWs, 1 and 0.5 ha, retained higher total amounts of P (i.e. orange and red colours) than the smaller CWs, 0.1 and 0.2. For instance, all except one 1.0-ha CW and about half of the 0.5-ha CWs were among the top 20 CWs with the highest total P retention, i.e. these CWs are coloured red or orange in Fig. 4a. However, comparison based on the area-specific P retention revealed a different pattern. The smaller CWs (0.1 and 0.2 ha) situated in arable land areas (Fig. 2) had the largest area-specific retention (i.e. these CWs are coloured red or orange in Fig. 4b), whereas the largest circles representing the largest CWs (1 ha) are now coloured mostly in yellow and green (Fig. 4b), indicating lower area-specific retention. The top 21 CWs with the highest area-specific P retention were smaller CWs. The first 0.5-ha CW on the list was in 22nd place and the first 1-ha CW in 34th place. Furthermore, it is clearly shown that regardless of the size, the CWs located in the forested areas in the upper parts of the catchment have lowest (blue) absolute (Fig. 4a) and area-specific retention (Fig. 4b).

**Fig. 5:**
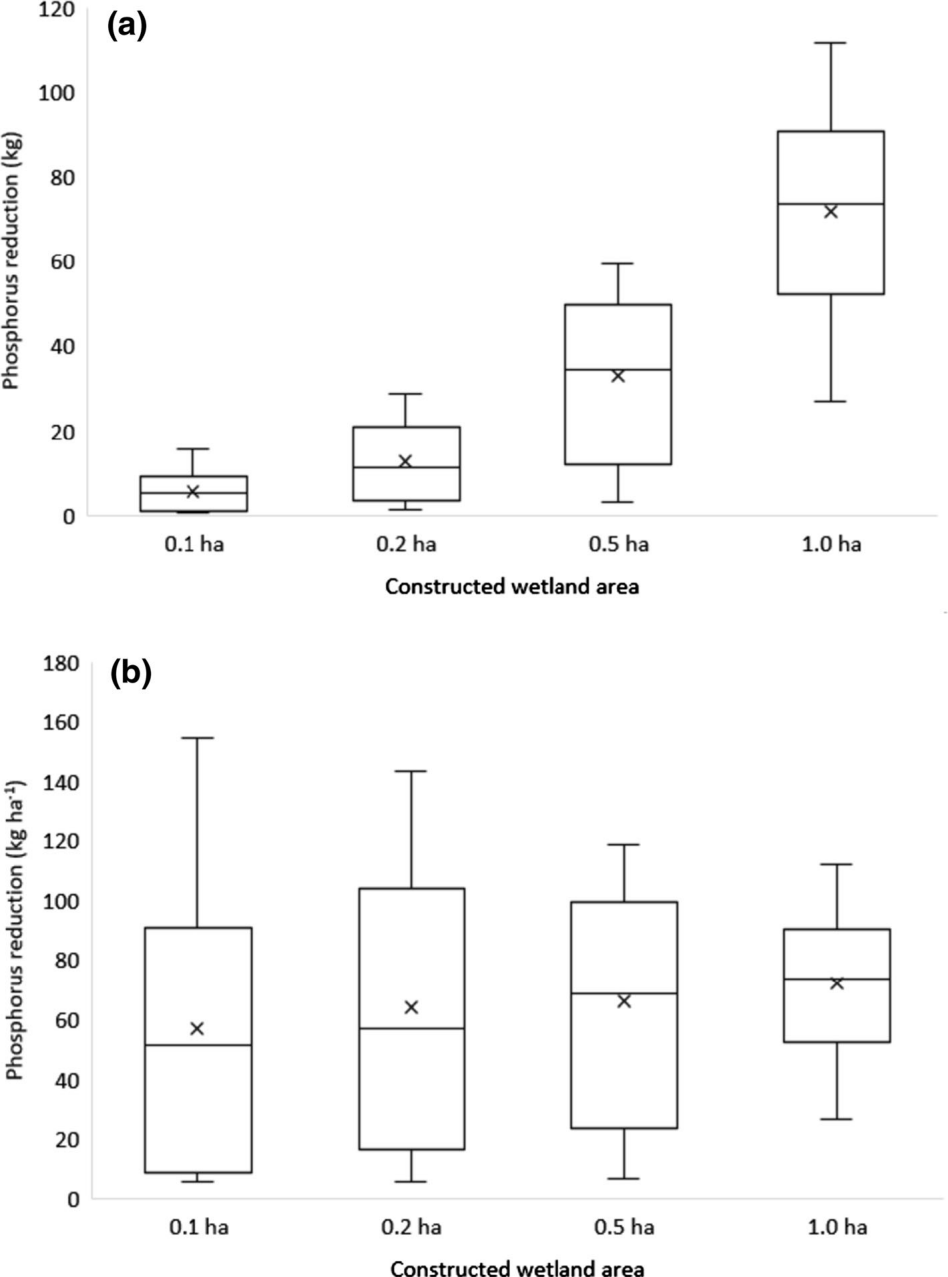
**a** Reduction in total phosphorus expressed as total reduction (kg) and **b** as area-specific reduction (kg ha^−1^) for four different size groups of constructed wetlands in Lillån catchment. Boxes show minimum, 25th, 50th (median), 75th percentile and maximum and mean value (*x*)

Potential TP retention showed a wide range of variation both within and between different size groups of CWs, both when expressed in absolute amounts of retained TP (kg, Fig. 5a) and per area CW (kg ha^−1^, Fig. 5b).

**Fig. 6:**
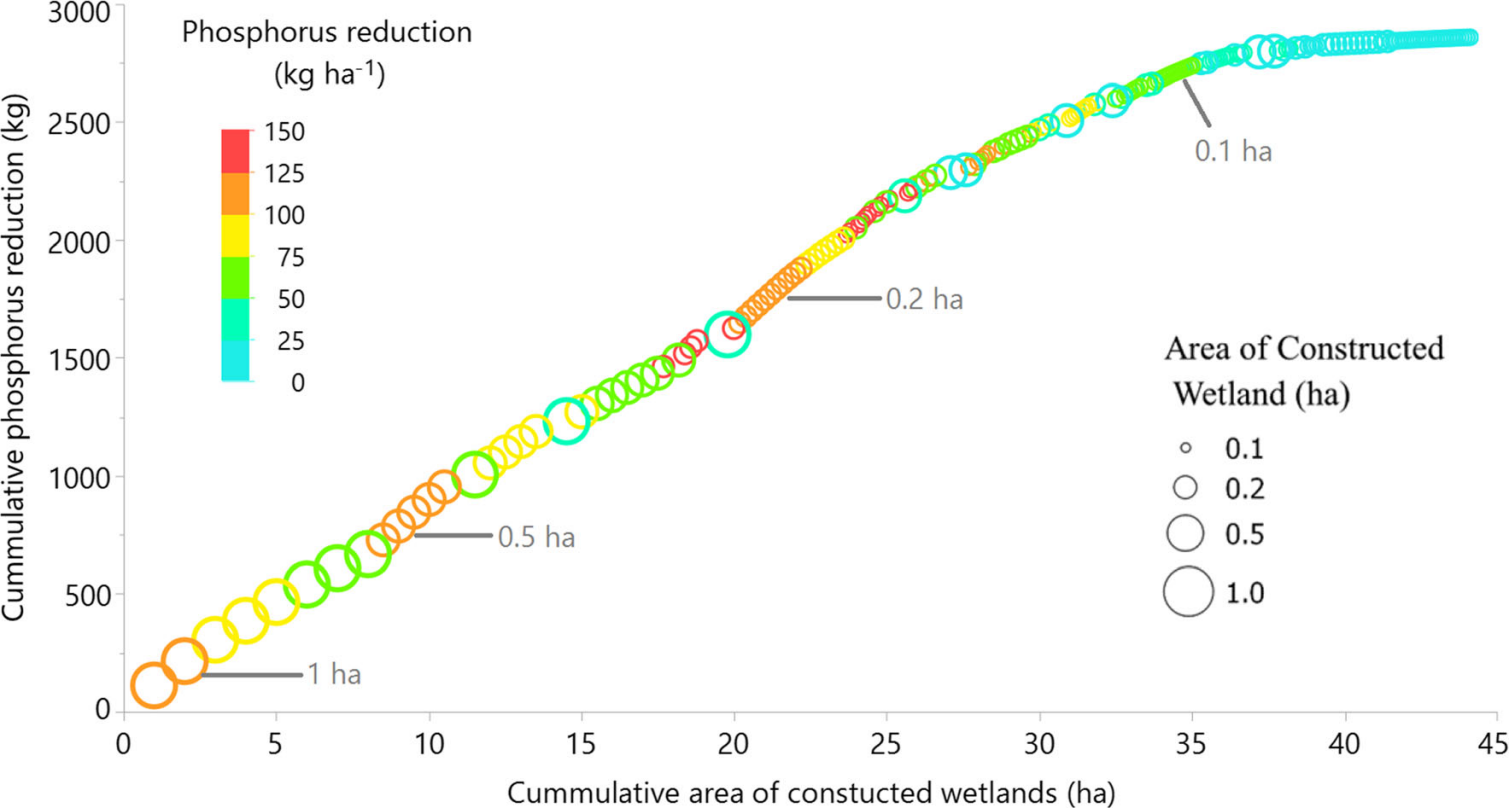
Cumulative modelled total phosphorus (TP) retention by constructed wetlands (CWs) in the study catchment, plotted against combined CW area of four different size groups. The CWs are ranked based on decreasing modelled TP retention. Circle size represents the area of the CW, whereas colours illustrate area-specific P retention (kg ha^−1^)

The largest (1.0-ha) CWs showed the highest absolute TP retention, with an average value of 72 kg and a median value very close to the average value (74 kg) (Fig. 5a). Average absolute TP retention increased with CW size, since smaller total amounts of TP reached smaller CWs due to smaller catchments draining to these. However, even when expressed per CW area (kg ha^−1^, Fig. 5b), the average retention was highest for the large 1.0-ha and 0.5-ha CWs. At the same time, the variability in modelled area-specific retention (kg ha^−1^) of TP within each group decreased with CW size. For instance, minimum and maximum were 27 and 112 kg ha^−1^, respectively, for the 1.0-ha group, whereas corresponding values for the 0.1-ha group were 5.5 and 155 kg ha^−1^. Consequently, both the least effective and the most effective CWs were found in the smallest size group, 0.1-ha (Figs. 5b and 6). Furthermore, there was a linear response in terms of TP reduction for a total CW area of approximately 35 ha in Lillån catchment, and thereafter the effect is much lower (Fig. 6).

## Discussion

We calculated the potential for TP retention in CWs, using a medium-size catchment in central Sweden as a case. Similar calculations have been performed previously, e.g. in an attempt to give guidance on formulation of river basin management plans to comply with the European Union Water Framework Directive (Gyllström et al. 2016). Based on rather coarse estimates, the potential area for P ponds and CWs in Lillån catchment needed to reach good ecological status is reported to be 43 and 214 ha, respectively (Gyllström et al. 2016). However, results presented by Kynkäänniemi (2014) indicated a similar response of P ponds and CWs to the hydraulic load, indicating that their area should also be similar. Thus today, even large wetlands are constructed using the same design as for P ponds, i.e. with a deeper initial part followed by a shallower vegetated area. Therefore P ponds and wetlands were treated identically in the present study. However, the total potential area for wetland construction based on the optimal HL of 100 m year^−1^ estimated in the present study was 44.1 ha, which is only 17% of the combined potential area (43 ha + 214 ha) estimated by Gyllström et al. (2016). In addition, some (~ 9 ha) of the potential CWs fulfilling the HL = 100 criterion (in total 44.1 ha) in this study were estimated to be very ineffective (Fig. 6), due to low P load as they were mostly located in forested areas. Based on Fig. 6, there is a linear response in terms of TP reduction for a total CW area of approximately 35 ha in the study catchment, and thereafter the effect is much lower. Moreover, CWs of different sizes may ‘compete’ for the same upstream area, and therefore it would be suboptimal to construct them all. For instance, if two separate tributaries in the catchment are suitable for two 0.5-ha CWs, constructing another 1-ha CW after the confluence of these tributaries would result in a lower retention effect, due to the reduced input to the downstream 1-ha CW. Hence, the potential areas of efficient CWs in Lillån catchment is even lower.

The wide variation in TP retention, in total amounts or expressed per area of CW, suggests a high potential for prioritisation based on placement of the CWs. The smallest CWs (0.1 ha) showed the highest variation in TP retention, ranging from 0.5 to 15.5 kg TP (~ 31-fold). Overall, depending on the land use and soil distribution in the catchment draining to each CW, the loads of TP reaching CWs varied most for the smallest catchments. Some of these upstream catchments were pure forest catchments with low P losses, whereas others were dominated by arable land and P loss-sensitive clay soils (Sandström et al. 2019), resulting in high P loads and, consequently, high P retention. Moving downstream, with larger CWs and larger catchment areas, the land use is more mixed, with smaller differences between land use distributions. As a consequence of this, variation in TP retention decrease with increasing CW size. However, even among the largest (1.0-ha) CWs and corresponding catchments, there were still large differences in TP retention, which ranged from 27 to 112 kg TP (fourfold). The importance of proper placement of potential CWs is illustrated by the following extreme values: The most efficient 0.2-ha CW retained more P (29 kg TP) than the least effective 1.0-ha CW (27 kg TP), despite being just one-fifth of the area, thereby saving more arable land for farming. Considering that the highest P retention variation was observed in the smaller CWs, targeting the optimal location seems to be most important for these CWs. The fourfold difference in P retention potential between the large (1.0-ha) CWs should be reason enough to consider optimisation of their location too, especially if there are limited resources available for new CWs. Possible prioritisation of CWs of different sizes also needs to take into account the construction costs. Several of the existing CWs in Lillån catchment (Water Information System Sweden 2020) are very large (4 to 29 ha) and thereby too large in relation to incoming hydraulic and nutrient loads to achieve high reduction efficiency.

As shown in Fig. 5b, the estimated area-specific minimum and maximum removal rate varied between 5.5 and 155 kg ha^−1^, both values for 0.1-ha CWs, with the median values ranging from 51 kg ha^−1^ (0.1-ha CWs) to 74 kg ha^−1^ (1-ha CWs). Elsewhere, Uusi-Kämppä et al. (2000) reported higher measured removal rate for CWs (median 630 kg ha^−1^), but rather comparable removal rate for ponds included in the study (median 70 kg ha^−1^). Additionally, in a large systematic review of published studies on constructed and restored wetlands, Land et al. (2016) reported variation in median removal rate between 6.3 kg ha^−1^ (for the strongest study quality category, category 3) and 29 kg ha^−1^ (for the moderate study quality category, category 2). These reports collected large number of individual studies in Nordic countries ((Uusi-Kämppä et al. 2000) and in the world (Land et al. 2016), with highly variable preconditions important for the functioning of CWs. Our study focuses on one catchment, Lillån, with, for Swedish conditions, moderate P losses. However, as the removal rate is depending on incoming P loads, scaling up here presented methodology to other catchments in Sweden and elsewhere, with considerably lower or higher P losses resulting in both lower and higher removal rates, which may be easier to compare with the results from the abovementioned studies.

The proposed modelling approach, combining high-resolution distributed modelling with export coefficients for different land uses, is a robust way to account for TP fluxes in a catchment. Modelled TP loads at monthly time steps for two different agricultural catchments of different sizes and patterns of soil distribution showed reasonable agreement with measured values (Fig. 3), although the modelled values sometimes overestimated P losses, by 14–31%. A possible explanation for this overestimation might be use of an average value for all arable crops instead of field-specific crops, as information on the latter was lacking. The approach has been tested elsewhere for an additional six objects, two arable fields (4.4 and 33.8 ha) and four agricultural catchments (range 740–1630 ha), in southern Sweden, covering contrasting soil textural distributions (from sand to clay soils) (Djodjic et al. 2019). Comparable results have been obtained in all tests, which indicate the robustness and applicability of the approach to a wide range of edaphic and climate conditions. In the present model application to Lillån catchment, the main focus was on spatial variations in TP load as a function of land use and soil distribution in the catchments of potential CWs. Although the model slightly overestimated TP loads in neighbouring catchments C6 and U8, the modelled results for C6 and U8 captured the spatial differences between these catchments. Therefore the estimated spatial variations in Lillån catchment should be considered reliable, at least for spatial comparisons at the relative scale.

Furthermore, the presented modelling approach makes it possible to estimate the optimal location for efficient future wetlands by accounting for possible changes in water flow and/or land cover distribution and management due to climatic or societal changes where altered hydraulic or nutrient loads to the CWs might require adjusted wetland area and placement. The proposed Nordic Bioeconomy Pathways with alternative agricultural and forest systems suggested by Rakovic et al. (2020), if properly quantified, can be used as input data to accordingly change calculations in the method proposed here. Indeed, as the set of attributes that characterise agricultural and forest management under these alternative possible futures are currently articulated to quantitative scenarios as inputs to process-based models (Rakovic et al. 2020), the same input data can be used both in the first step (e.g. change in mean annual flow) and in the second step (e.g. change of export coefficients produced by ICECREAM) of the methodology presented here to adjust placement and size of CWs. For example, the currently largely redundant wetlands and ponds in the upper forested parts of the catchment may become more important to retain increased loads from more intensively exploited and fertilised forests.

## Conclusions and future implications

As investment in CWs is predicted to continue due to the need to reduce P delivery to water recipients, increasing the P retention efficiency of CWs by optimising their placement is a plausible option to increase the cost-efficiency of CW installation. Wetland size in relation to its upstream area determines the annual hydraulic load, and can be used to calculate the total potential area for CWs in a given catchment and to determine proper placement of CWs of various sizes to enable maximum P retention. This study showed that the potential area for CWs is much lower than estimated previously in a specific catchment. It also showed that P retention in CWs will differ based on the incoming P loads, which in turn are the result of land use and soil distribution in the upstream area of each CW. By accounting for this difference in P loads through use of robust models based on land use- and soil texture-specific export coefficients, we revealed great spatial variability in potential P retention. We found that the smallest CWs (0.1 ha) experienced the highest variation in P retention, depending on the dominant land use/soil texture in the catchment. As CW size and the corresponding catchment increase, the catchment becomes more mixed, which reduces the variation in P retention. However, there were large spatial variation in P retention in all four size groups of CWs (0.1–1.0 ha) and regardless of size, CWs in forested areas had lowest P retention showing the possible benefits of targeted placement of CWs.

The proposed modelling approach could be further improved by accounting for the field-specific crop distribution, if such data become available. In addition, the correlation between hydraulic load and annual P accumulation is currently based on limited number of wetlands (*n* = 8), and should be verified/modified based on a higher number of CWs. Using the same modelling approach, the results presented here could be scaled up to cover the majority of arable land in Sweden, or elsewhere if similar input data are available, but also adjusted for alternative future scenarios and possible changes in water flow and/or land cover distribution and management.

## References

[CR1] Braskerud BC, Tonderski KS, Wedding B, Bakke R, Blankenberg AGB, Ulen B, Koskiaho J (2005). Can constructed wetlands reduce the diffuse phosphorus loads to eutrophic water in cold temperate regions?. Journal of Environmental Quality.

[CR2] Carpenter SR, Caraco NF, Correll DL, Howarth RW, Sharpley AN, Smith VH (1998). Nonpoint pollution of surface waters with phosphorus and nitrogen. Ecological Applications.

[CR4] Djodjic F, Markensten H (2018). From single fields to river basins: Identification of critical source areas for erosion and phosphorus losses at high resolution. Ambio.

[CR3] Djodjic F, Elmquist H, Collentine D (2018). Targeting critical source areas for phosphorus losses: Evaluation with soil testing, farmers’ assessment and modelling. Ambio.

[CR5] Djodjic F, Markensten H, Sandström S, Widén Nilsson E, Persson K, Lindsjö A, Johnsson H, Mellander P-E, Leach S, Burgess E (2019). Combining high-resolution spatially distributed models with export coefficients produced by field-scale process-oriented model. Catchment science 2019 - Achieving quality water in diverse and productive agricultural landscapes under a changing climate.

[CR6] Ejhed, H., E. Widén-Nilsson, J. Tengdelius-Brunell, and J. Hytteborn. 2016. Nutrient loads to the Swedish marine environment in 2014—Swedish contribution to HELCOM:s Sixth Polution Load Compilation. Swedish Agency for Marine and Water Management, Report 2016:2, Göteborg, Sweden (in Swedish, English summary).

[CR7] Fisher J, Acreman MC (2004). Wetland nutrient removal: A review of the evidence. Hydrology and Earth System Sciences.

[CR8] Gyllström, M., M. Larsson, J. Mentzer, J.F. Petersson, M. Cramér, P. Boholm, and E. Witter. 2016. Countermeasures against eutrophication to achieve god ecological status - Base for water authorities’ proposals for abatement program Länsstyrelsen i Västmanlands län, Länsstyrelsens rapportserie 2016:19, Västerås, Sweden (in Swedish).

[CR9] Johannesson KM, Kynkäänniemi P, Ulén B, Weisner SEB, Tonderski KS (2015). Phosphorus and particle retention in constructed wetlands—A catchment comparison. Ecological Engineering.

[CR10] Johnsson, H., M. Larsson, A. Lindsjö, K. Mårtensson, K. Persson, and G. Torstensson. 2008. Leaching of nutrients from Swedish arable land. Swedish Environmental Protection Agency, Rapport 5823, Stockholm, Sweden (in Swedish, English summary).

[CR11] Johnsson, H., K. Mårtensson, A. Lindsjö, K. Persson, Y. Andrist Rangel, and K. Blombäck. 2016. Leaching of nutrients from Swedish arable land—Calculations of normalized losses for nitrogen and phosphorus 2013. SMED, Rapport 189, Norrköping, Sweden (in Swedish).

[CR12] Johnsson, H., K. Mårtensson, A. Lindsjö, K. Persson, Y.A. Rangel, and K. Blombäck. 2019. Leaching of nutrients from Swedish arable land—Calculations of normalized losses for nitrogen and phosphorus 2016. SMED, Rapport 5, Norrköping, Sweden (in Swedish).

[CR13] Koskiaho, J. 2006. Retention performance and hydraulic design of constructed wetlands treating runoff waters from arable land. PhD thesis. Oulu, Finland University of Oulu.

[CR14] Kyllmar K, Forsberg LS, Andersson S, Mårtensson K (2014). Small agricultural monitoring catchments in Sweden representing environmental impact. Agriculture, Ecosystems & Environment.

[CR15] Kynkäänniemi, P. 2014. Small wetlands designed for phosphorus retention in Swedish agricultural areas. PhD thesis. Uppsala Swedish University of Agricultural Sciences.

[CR16] Kynkäänniemi P, Ulén B, Torstensson G, Tonderski KS (2013). Phosphorus retention in a newly constructed wetland receiving agricultural tile drainage water. Journal of Environmental Quality.

[CR17] Land M, Granéli W, Grimvall A, Hoffmann CC, Mitsch WJ, Tonderski KS (2016). How effective are created or restored freshwater wetlands for nitrogen and phosphorus removal? A systematic review. Environmental Evidence.

[CR18] Lantmäteriet. 2014. Produktbeskrivning: GSD-Höjddata, grid 2 + Lantmäteriet, GSD Geografiska Sverige Data, Gävle, Sweden (in Swedish).

[CR19] Larsson MH, Persson K, Ulen B, Lindsjo A, Jarvis NJ (2007). A dual porosity model to quantify phosphorus losses from macroporous soils. Ecological Modelling.

[CR20] Nash JE, Sutcliffe JV (1970). River flow forecasting through conceptual models part I—A discussion of principles. Journal of Hydrology.

[CR21] O’Geen AT, Budd R, Gan J, Maynard JJ, Parikh SJ, Dahlgren RA, Sparks DL (2010). Chapter one—Mitigating nonpoint source pollution in agriculture with constructed and restored wetlands. Advances in Agronomy.

[CR22] Piikki K, Söderström M (2019). Digital soil mapping of arable land in Sweden—Validation of performance at multiple scales. Geoderma.

[CR100] Rakovic, J., M.N. Futter, K. Kyllmar, K. Rankinen, M.I. Stutter, J. Vermaat, and D. Collentine. 2020. Nordic Bioeconomy Pathways: Future narratives for assessment of water-related ecosystem services in agricultural and forest management. *Ambio*. (This issue). 10.1007/s13280-020-01389-7. 10.1007/s13280-020-01389-7 PMC7487143 32920768

[CR24] Sandström S, Futter MN, Kyllmar K, Bishop K, O’Connell DW, Djodjic F (2019). Particulate phosphorus and suspended solids losses from small agricultural catchments: Links to stream and catchment characteristics. Science of the Total Environment.

[CR25] Schmitz O, Karssenberg D, van Deursen WPA, Wesseling CG (2009). Linking external components to a spatio-temporal modelling framework: Coupling MODFLOW and PCRaster. Environmental Modelling & Software.

[CR26] Sharpley AN, Bergström L, Aronsson H, Bechmann M, Bolster C, Börling K, Djodjic F, Jarvie H (2015). Future agriculture with minimized phosphorus losses to waters: Research needs and direction. Ambio.

[CR27] Sharpley AN, Kleinman PJA, Jordan P, Bergstrom L, Allen AL (2009). Evaluating the success of phosphorus management from field to watershed. Journal of Environmental Quality.

[CR28] Sidemo-Holm W, Smith HG, Brady MV (2018). Improving agricultural pollution abatement through result-based payment schemes. Land Use Policy.

[CR29] SLU. 2018. Monitoring nutrient losses from arable land. https://www.slu.se/en/departments/soil-environment/research/water-quality-management/monitoring-nutrients/. Accessed 29 Aug 2017.

[CR30] Swedish Environment Protection Agency. 2019. Environmental objectives—Annual follow up on fullfilement of Swedish national environmental objectives 2019. Swedish Environment Protection Agency, Report 6890, Stockholm, Sweden (in Swedish).

[CR31] Söderström, M., and K. Piikki. 2016. Digital soil map—Detailed mapping of soil texture in the topsoil of the arable land. Swedish University of Agricultural Sciences, Technical report 37, Skara, Sweden (In Swedish).

[CR32] Thomas IA, Mellander PE, Murphy PNC, Fenton O, Shine O, Djodjic F, Dunlop P, Jordan P (2016). A sub-field scale critical source area index for legacy phosphorus management using high resolution data. Agriculture, Ecosystems & Environment.

[CR33] Tonderski KS, Arheimer B, Pers CB (2005). Modeling the impact of potential wetlands on phosphorus retention in a Swedish catchment. Ambio.

[CR34] Ulén B, Geranmayeh P, Blomberg M, Bieroza M (2019). Seasonal variation in nutrient retention in a free water surface constructed wetland monitored with flow-proportional sampling and optical sensors. Ecological Engineering.

[CR35] Uusi-Kämppä J, Braskerud B, Jansson H, Syversen N, Uusitalo R (2000). Buffer zones and constructed wetlands as filters for agricultural phosphorus. Journal of Environmental Quality.

[CR36] Water Information System Sweden. 2020. Proposed and implemented measures for the water body: Lillån, Kvarnbrobäcken, Hovgårdsbäcken, Åbylundsbäcken, Tomtabäcken. https://viss.lansstyrelsen.se/Waters.aspx?waterMSCD=WA49319905#pagemodule25. Accessed 3 Mar 2020.

[CR37] Weisner SEB, Johannesson K, Thiere G, Svengren H, Ehde PM, Tonderski KS (2016). National Large-Scale Wetland Creation in Agricultural Areas—Potential versus realized effects on nutrient transports. Water.

